# Revisiting methods for modeling longitudinal and survival data: Framingham Heart Study

**DOI:** 10.1186/s12874-021-01207-y

**Published:** 2021-02-10

**Authors:** Julius S. Ngwa, Howard J. Cabral, Debbie M. Cheng, David R. Gagnon, Michael P. LaValley, L. Adrienne Cupples

**Affiliations:** 1 Department of Biostatistics, School of Public Health, Boston University, 801 Massachusetts Ave, CT 3rd Floor, Boston, MA 02118 USA; 2 Department of Biostatistics, Johns Hopkins Bloomberg School of Public Health, 615 North Wolfe St E3009, Baltimore, MD 21205 USA; 3 National Heart, Lung, and Blood Institute’s Framingham Heart Study, Framingham, MA 01702 USA

**Keywords:** Joint longitudinal and survival model, Cox model, Two-step approach, Mixed effect modeling, Time dependent covariate models, Weibull distribution, Residual variance

## Abstract

**Background:**

Statistical methods for modeling longitudinal and time-to-event data has received much attention in medical research and is becoming increasingly useful. In clinical studies, such as cancer and AIDS, longitudinal biomarkers are used to monitor disease progression and to predict survival. These longitudinal measures are often missing at failure times and may be prone to measurement errors. More importantly, time-dependent survival models that include the raw longitudinal measurements may lead to biased results. In previous studies these two types of data are frequently analyzed separately where a mixed effects model is used for the longitudinal data and a survival model is applied to the event outcome.

**Methods:**

In this paper we compare joint maximum likelihood methods, a two-step approach and a time dependent covariate method that link longitudinal data to survival data with emphasis on using longitudinal measures to predict survival. We apply a Bayesian semi-parametric joint method and maximum likelihood joint method that maximizes the joint likelihood of the time-to-event and longitudinal measures. We also implement the Two-Step approach, which estimates random effects separately, and a classic Time Dependent Covariate Model. We use simulation studies to assess bias, accuracy, and coverage probabilities for the estimates of the link parameter that connects the longitudinal measures to survival times.

**Results:**

Simulation results demonstrate that the Two-Step approach performed best at estimating the link parameter when variability in the longitudinal measure is low but is somewhat biased downwards when the variability is high. Bayesian semi-parametric and maximum likelihood joint methods yield higher link parameter estimates with low and high variability in the longitudinal measure. The Time Dependent Covariate method resulted in consistent underestimation of the link parameter. We illustrate these methods using data from the Framingham Heart Study in which lipid measurements and Myocardial Infarction data were collected over a period of 26 years.

**Conclusions:**

Traditional methods for modeling longitudinal and survival data, such as the time dependent covariate method, that use the observed longitudinal data, tend to provide downwardly biased estimates. The two-step approach and joint models provide better estimates, although a comparison of these methods may depend on the underlying residual variance.

**Supplementary Information:**

The online version contains supplementary material available at 10.1186/s12874-021-01207-y.

## Background

Statistical methods for modeling longitudinal and time-to-event data has received much attention recently in medical research and is becoming increasingly useful. A common objective in this research is to characterize the relationship between the longitudinal response and the time-to-event [1, 2]. Typical settings where these may occur include HIV studies in which baseline characteristics are recorded and immunological measures such as CD4+ lymphocyte counts or viral load are measured repeatedly to assess patients’ health until HIV conversion [3]. We consider data from the Framingham Heart Study in which lipid measurements and myocardial infarction (MI) data were collected over a period of 26 years. The Framingham Heart Study (http://www.framinghamheartstudy.org/) is a well-known longitudinal study that identifies potential risk factors for the development of cardiovascular disease. In this study high-density lipoprotein (HDL), low density lipoprotein (LDL) and triglycerides (TG) were measured at generally comparable time intervals over the 26-years. Time to MI was also recorded for each participant, although some subjects were censored by the end of the study period or due to death from other causes. We assess methods that characterize associations between the longitudinal lipid measures and time to MI with an emphasis on precise estimation of the parameters linking longitudinal risk factors with time to MI.

There is extensive literature and a wide range of statistical packages for jointly modeling longitudinal and survival data. Some recent work includes those of Brown and Ibrahim [4]; Zeng and Cai [5]; Tseng, Hsieh, and Wang [6]; Ye, Lin, and Taylor [7]; Ibrahim, Chu, and Chen [8]; Rizopoulos [9], among others. Tsiatis and Davidian [2] provide a comprehensive overview of earlier articles addressing a number of methods for jointly modeling longitudinal and survival data. These articles include work on joint models by Robins and Tsiatis [10]; DeGruttola and Tu [11]; Tsiatis, DeGruttola, and Wulfsohn [12]; LaValley and Degruttola [13]; Faucett and Thomas [14]; Wulfsohn and Tsiatis [15]; Wang and Taylor [16]; Xu and Zeger [17]. Sweeting and Thompson [18] provide a comparison of a shared random effects model with a naïve time-dependent covariate model and a two-stage joint model for modelling the association between the longitudinal process and the time-to-event outcome. They conducted a simulation study to contrast these three approaches in their ability to estimate the true association between a longitudinal process and the event hazard. In their simulations they assumed a constant baseline hazard model to simulate a relatively rare event with a modest correlation between the longitudinal and survival process [18]. Our paper builds upon the Sweeting and Thompson paper and implements a new data generation scheme, assuming a Weibull distribution for the survival process and considers a wide range of scenarios for the event rates and the residual variances.

Our main objectives in this paper are to (i) evaluate a Bayesian semi-parametric joint model (BSJM) and a maximum likelihood joint model approach (MLA) that link longitudinal trajectories to survival and (ii) compare these joint maximum likelihood methods with the Two-Step Approach (TSA), and the Cox Time Dependent Covariate Model (TDCM) [4, 12, 15, 19]. In these methods the BSJM and MLA maximize the joint likelihood of the longitudinal and survival data. In the TSA, random effects are estimated separately in the first stage and the predicted longitudinal measures are then substituted into the second stage survival analysis. The argument in favor of the joint model has been the efficient use of the data as the survival information goes into modeling the longitudinal process and vice versa. The TDCM implements time varying covariate approaches in which the observed longitudinal measures are applied in the survival model.

## Methods

In this section, we describe the methods for jointly modeling longitudinal data and survival data using a joint likelihood. In such modeling, the main focus may be on the longitudinal component, the survival component, or both, depending on the objectives of the studies. When the focus is on one aspect, the other component is then secondary; so its parameters may be viewed as nuisance parameters [20]. Our goal is to characterize the relation between the time-to-event (primary) outcome and the longitudinal measures (secondary), adjusting for covariates in the model.

### Joint likelihood model

We consider a joint likelihood model similar to that of Brown and Ibrahim [4], which links the longitudinal trajectories of each subject to survival data. The longitudinal responses, *Y*_*i*,_ are linked to the time-to-event model using a Cox proportional hazard model [21]. For each individual, *i*, we let *S*_*i*_ be the survival time and *C*_*i*_ censoring time, with *T*_*i*_ the observed survival time. Due to censoring we observe *T*_*i*_ =  *min* (*S*_*i*_, *C*_*i*_). Let *δ*_*i*_ = *I*(*S*_*i*_ ≤ *C*_*i*_), denote the event indicator:

$$ {\delta}_i=\left\{\begin{array}{c}1\  if\ {S}_i\le {C}_i\\ {}0\  if\ {S}_i>{C}_i\ \end{array}\right\} $$

The joint likelihood for subject *i* can be constructed as a product of the longitudinal model and survival model conditional on the longitudinal measures.

$$ f\left({Y}_i,{t}_i,{\delta}_i\right)=f\left({Y}_i\right)\ast f\left({t}_i,{\delta}_i|{Y}_i\right) $$

We begin with the likelihood model for the longitudinal measures and implement a random effects approach that models the longitudinal measures with possible measurement errors. Each subject has *m*_*i*_ measures denoted by *Y*_*ij*_, *j* = 1, 2, …, *m*_*i*_, where *Y*_*ij*_ represents the observed outcome for the *i*^*th*^ subject at the *j*^*th*^ time point. We denote *Y*_*ij*_^∗^ as the true unobserved measure value such that:

1 $$ {Y}_{ij}\left({t}_{ij}\right)={Y_i}^{\ast}\left({t}_{ij}\right)+{\epsilon}_{ij},{\epsilon}_{ij}\sim N\left(0,{\sigma}^2\right) $$

${Y}_{ij}^{\ast }$ is also known as the trajectory function. Throughout this paper we assume that *Y*_*ij*_^∗^ = *Y*_*i*_^∗^(*t*_*ij*_). The trajectory can be modeled in a linear form [4] or quadratic form [12], or spline, and other time series forms can be implemented to capture the trajectory of the longitudinal measures but the trade-off is the complexity and interpretation of the model. These longitudinal measures are often missing at failure times and may be prone to measurement error. Including the raw longitudinal measurements in analysis may lead to bias if the measures are related to the censoring process [1].

In this paper we use a linear mixed effects model (LME), which allows individual or subject-specific inference following the approach of Laird and Ware [22], to fit data from longitudinal response processes.

2 $$ E\left(\ {Y}_{ij}\right)={Y}_i^{\ast}\left({t}_{ij}\right)={U}_{i\mathbf{1}}+{U}_{i\mathbf{2}}\ast {t}_{ij} $$

Here *U*_*i*1_ and *U*_*i*2_ are random effects, representing the subject-specific intercepts and slopes, and are usually assumed to be multivariate normally distributed. The variable *t*_*ij*_ represents the times for the *i*^*th*^ subject at the *j*^*th*^ time at which the longitudinal measures are recorded during the follow-up period.

For the survival model we consider the Cox model that links the time-to-event data and the longitudinal trajectories through the hazard function. For each individual, *i* = 1, 2, …, *n*, we let *S*_*i*_ denote the survival or event time and *C*_*i*_ denote the censoring time, respectively. We assume that the censoring process is random or non-informative.

For individual, *t*_*i*_ denotes the failure time, which may be right censored. The Cox model that links the time-to-event outcome and the longitudinal trajectories through the hazard function can be written as:

3 $$ {h}_i(t)={h}_{\mathbf{0}}(t)\exp \left\{{Y}_i^{\ast }(t)\gamma +{X_i}^T\alpha \right\} $$

The parameter *γ* is a scalar (link) parameter that links the predicted longitudinal trajectories to the hazard function; *α* is a vector of unknown parameters for the time independent covariate measures *X*_*i*_^*T*^; *h*_0_(*t*) is the baseline hazard function. From (3) one can generate a Cox partial likelihood [21] from which statistical inferences can be derived if ${Y}_i^{\ast }$ were observed:

4 $$ {L}_p^{\ast }=\prod \limits_{i=1}^n{\left\{\frac{\mathit{\exp}\left({Y}_i^{\ast}\left({t}_i\right)\gamma +{X_i}^T\alpha \right)}{\sum \limits_{k=1}^n\mathit{\exp}\left({Y}_k^{\ast}\left({t}_i\right)\gamma +{X_i}^T\alpha \right)I\left({t}_k\ge {t}_i\right)}\right\}}^{\delta_i} $$

In (4) two assumptions are made: (i) survival times are independent and without ties; (ii) the longitudinal measures are available at each event time for all individuals. Assumption (ii) is often not true as covariate measurement times may not coincide with event times, leading to missing data in time-dependent covariates in the survival model. Such missingness may be assumed to be ignorable or MAR where the missingness is not related to the missing values that would be observed [23]. The last-value-carried-forward (LVCF) method for missing longitudinal measures has been widely used to impute the missing covariates, but may lead to bias in the estimates [1].The hazard function for the survival component (3) given the longitudinal trajectory function yields:

$$ f\left({t}_i,{\delta}_i\right)\propto f{\left({t}_i\right)}^{\delta_i}S{\left({t}_i\right)}^{1-{\delta}_i}=h{\left({t}_i\right)}^{\delta_i}S\left({t}_i\right), where\ S(t)=\mathit{\exp}\left(-{\int}_0^th(u) du\right) $$

5 $$ f\left({t}_i,{\delta}_i|{Y_i}^{\ast },{X}_i\right)={h}_0{\left({t}_i\right)}^{\delta_i}\exp \left\{{\delta}_i\left({Y}_i^{\ast}\left({t}_i\right)\gamma +{X_i}^T\alpha \right)\right\}\ast \mathit{\exp}\left\{-{\int}_0^{t_i}{h}_0(u)\exp \left[{Y}_i^{\ast }(u)\gamma +{X_i}^T\alpha \right] du\right\} $$

Statistical inference based on the above likelihood function is potentially computationally intensive. A non-adaptive Gauss-Kronrod integration can be employed to numerically calculate the integral [24]. The maximum likelihood parameter estimates can be obtained using the Expectation–Maximization (EM) algorithm and Newton-Raphson approximation [25]. Or a Monte Carlo Markov Chain (MCMC) approach can be implemented in a Bayesian framework [14]. Tsiatis and Davidian [2] applied a different approach to the issue by proposing a conditional score that is also efficient and yields consistent and asymptotically normal estimators.

#### Bayesian semi-parametric joint model (BSJM)

Faucett and Thomas [14] applied a Bayesian approach to estimate the parameters of the longitudinal process (σ^2^, mean and covariance of *U*_*i*1_ and *U*_*i*2_) and the proportional hazard model (λ_0_, γ and *α*) in the joint likelihood framework via MCMC. They specified non-informative priors for the parameters to obtain results similar to the maximum likelihood estimates. Wang and Taylor [16] applied a similar approach to model the survival component and used a more flexible approach to the longitudinal part by incorporating a stochastic process into the longitudinal trajectory. Brown and Ibrahim [4] considered a semi-parametric Bayesian joint model in which they relax the distributional assumptions for the longitudinal model using Dirichlet process priors on the random effect parameters.

We apply a Bayesian approach for jointly modeling longitudinal and survival data similar to that of Brown and Ibrahim [4]. For the survival model, we specify normal priors for the parameters *γ* and *α*. We use MCMC methods to obtain posterior distributions of the parameters given the data. For the longitudinal component, we consider the LME model in which we assume prior distributions for the mean parameters, with variance components. From (1) and (2) we have that:

$$ {Y}_{ij}\left({t}_{ij}\right)={Y_{ij}}^{\ast}\left({t}_{ij}\right)+{\epsilon}_{ij};{Y}_{ij}^{\ast }={U}_{i1}+{U}_{i2}\ast {t}_{ij};i=1,2,\dots, n;j=1,2,\dots, {m}_i $$

$$ {\epsilon}_{ij}\sim N\left(0,{R}_i\right) $$

$$ {Y}_{ij}\mid {U}_i\sim N\left({Y}_{ij}^{\ast },{V}_i\right),{V}_i={Z}_iG{Z_i}^{\prime }+{R}_i,{R}_i={\sigma}^2{I}_{m_i} $$

We model the longitudinal trajectories using a linear growth curve model with *U*_*i*1_ and *U*_*i*2_ representing the random intercepts and slopes. *G* denotes the covariance matrix of the random effects and *Z*_*i*_ is a diagonal matrix of the longitudinal time points. A subject *i* ′ *s* contribution to the joint likelihood function can be written as:

6 $$ f\left({Y}_i,{t}_i,{\delta}_i\right)={h}_{\mathbf{0}}{\left({t}_i\right)}^{\delta_i}\mathit{\exp}\left\{{\delta}_i\left({Y}_i^{\ast}\left({t}_i\right)\gamma +{X_i}^T\alpha \right)\right\}\ast \mathit{\exp}\left\{-{\int}_{\mathbf{0}}^{t_i}{h}_{\mathbf{0}}(u)\mathit{\exp}\left[{Y}_i^{\ast }(u)\gamma +{X_i}^T\alpha \right] du\right\}\ast $$

$$ \frac{1}{{\left(2\pi {\sigma}^2\right)}^{\frac{m_i}{2}}}\mathit{\exp}\left\{-\frac{1}{2{\sigma}^2}\sum \limits_{j=1}^{m_i}{\left({Y}_{ij}-{Y}_{ij}^{\ast}\right)}^2\right\} $$

A typical approach assumes a constant hazard ratio for the baseline hazard and normal prior distributions for the random effects and the unknown regression parameter (*α*). The variance-covariance matrix (*V*) is assigned a Wishart distribution through a precision matrix P:

$$ P={V}^{-1}\sim Wishart\left({Q}^{-1},v\right) $$

and

$$ {\sigma}^2\sim IG\left(a,b\right) $$

In the Wishart distribution, *Q* denotes the scale matrix and *v* denotes the degrees of freedom. *IG* represents inverse gamma distribution with shape *a* and scale parameter *b*.

We employed R and WinBUGS to obtain parameter estimates and credible intervals from the posterior distribution of the Bayesian modeling using standard Gibbs sampling MCMC methods [26, 27]. Once the model, data and initial values are specified in WinBUGS, the parameters can be monitored until convergence is attained. At the end of the MCMC process we obtain plots and diagnostic statistics to assess convergence in the parameters. One shortcoming of the BSJM is the computing time involved in estimating the parameters.

#### Maximum likelihood approach (MLA)

The maximum likelihood approach for jointly modeling longitudinal and survival data was described by Rizopoulos [9]. This method, employing the same general approach as Wulfsohn and Tsiatis [15], implements a shared parameter model for the joint modeling of longitudinal responses and time-to-event data.

The joint distribution for the longitudinal and survival model can be written as:

7 $$ f\left({Y}_i,{T}_i,{\delta}_i\right)=\int f\Big({Y}_i\left|{U}_i\right)\ast \left\{{h}_i{\left({T}_i|{U}_i\right)}^{\delta_i}S\left({T}_i|{U}_i\right)\right\}\ast f\left({U}_i\right)d{U}_i $$

where *S*(*T*_*i*_| *U*_*i*_) and *h*_*i*_(*T*_*i*_| *U*_*i*_) represent the survival function and the hazard function respectively conditional on the random effects, and *f*(*Y*_*i*_, *T*_*i*_, *δ*_*i*_) is the density function. The distributional form in (7) assumes that given the random effects, the longitudinal measures are independent of the time-to-event outcome and are independent of each other. The true unobserved values of the longitudinal measures $\left({Y}_{ij}^{\ast }(t)\right)$ are associated with the event outcome (*T*_*i*_) through the hazard function:

$$ {h}_i\left(t|{Y}_i(t)\right)={h}_0(t)\mathit{\exp}\left\{{Y}_{ij}^{\ast }(t)\gamma +{X_i}^T\alpha \right\} $$

Parameter estimates, from (7), can be obtained using the JM package in R. The package fits a shared parameter model for the joint modeling of normally distributed longitudinal responses and event time under the likelihood approach. The maximum likelihood estimation for joint models is based on the maximization of the log-likelihood corresponding to the joint distribution of the time-to-event and longitudinal outcomes [9]. The maximization is challenging as the integral of the survival function has no analytic solution. Following Rizopoulos, we implemented a Weibull model using Gauss-Hermite integration to approximate the integral. In the estimation process a hybrid optimization approach is employed that starts with EM and then continues with direct maximization. The procedure for the EM algorithm uses a fixed number of iterations and if convergence is not achieved it switches to a quasi-Newton algorithm until convergence is attained.

### Two step approach (TSA)

In the TSA, the parameters of the longitudinal process are estimated separately, and the estimated random effects are substituted directly into the survival model. This approach was first implemented by Tsiatis [12] in which a linear mixed effect model is fit to the longitudinal measures and the fitted values are inserted into the Cox Proportional Hazard model in the second stage as time dependent covariate measures. Ye, Lin, and Taylor [7] proposed two approaches for modeling the TSA called risk set regression calibration (RRC) and ordinary regression calibration (ORC). In the first approach the LME model is fit using the observed longitudinal data only among subjects with the event. This approach can be implemented if the longitudinal trajectories of subjects who experienced the event may be different from those who did not.

In the second approach the LME model is fit using observed longitudinal data from all subjects. In the first step the longitudinal process is estimated using the LME model in (1) and (2) and estimates of the random effects are used to obtain predicted values of the longitudinal measures at event times. In the second step the predicted longitudinal measures are used in the Cox Model to estimate the hazard for the event. The variance estimates for the parameters are obtained from the observed information of the partial likelihood function. See eq. (3). *X*_*i*_ represents the time independent covariate measures in the model and the ${Y}_i^{\ast }(t)$ represents the predicted values of the longitudinal measures at event time *t*. The link parameter (*γ*) in this approach can be interpreted as the association between the longitudinal measures at event time and the survival time. The estimation and inference for the hazard model (3) can be performed by using the partial likelihood theory proposed by Cox [21].

The main advantage of this approach is that it is simple and can be implemented using existing statistical packages. Tsiatis et al. [12] argue that a repeated measures random effects model for the covariate process is superior to naive methods where one maximizes the partial likelihood of the Cox using the observed covariates values. Ye, Lin, and Taylor [7] argue that there are two main disadvantages of a simple TSA; 1) it may provide biased estimates especially when the longitudinal process and the survival process are strongly associated; and 2) it does not incorporate the uncertainty of estimation in the first stage into the second stage, possibly leading to under-estimation of the standard errors. We evaluate several scenarios to assess the validity of these assumptions using simulation studies.

### Time dependent covariate modeling (TDCM)

A time dependent explanatory variable is one whose value for any given subject may change over the period of time that the subject is observed [28]**.** The TDCM employs the observed longitudinal measures to predict an event. Therneau and Grambsch [19] considered a well-known example of TDCM using the Stanford Heart Transplant Program. Data for a subject is presented as multiple observations, each of which applies to an interval of observation. A proportional hazards model is often used to analyze covariate information that change over time. The hazard may be thought of as being proportional to the instantaneous probability of an event at a particular time [21].

We consider sample of size n, consisting of [*T*_*i*_, *δ*_*i*_, [*Y*_*ij*_(*t*), 0 ≤ *t* ≤ *T*_*i*_], *i* = 1, 2, …, *n*], where *T*_*i*_ is the time-to-event for the *i*^*th*^ subject, *δ*_*i*_ is the event indicator. The vector *Y*_*ij*_(*t*) = [*Y*_*i*1_(*t*), *Y*_*i*2_(*t*), …, *Y*_*ig*_(*t*)]^*T*^ is a set of observed longitudinal measures, and *m*_*i*_ ≤ *g* is the number of times intervals for the *i*^*th*^ subject and *g* is the maximum.

$$ {\delta}_i=\left\{\begin{array}{c}1\  if\ {T}_i\le {C}_i\\ {}0\  if\ {T}_i>{C}_i\ \end{array}\right\} $$

The hazard for this model at time t can be written as:

8 $$ h(t)={h}_0(t)\ast \mathit{\exp}\left(\beta {Y}_i(t)+{X_i}^T\alpha \right)={h}_0(t)\ast \mathit{\exp}\left(\sum \limits_{k=1}^{m_i}{\beta}_k{Y}_{ik}(t)+{X_i}^T\alpha \right) $$

The vector ${Y}_{ij}(t)={\left[{Y}_{i1}(t),\dots, {Y}_{i{m}_i}(t)\right]}^T$ is a set of covariates and *m*_*i*_ is the number of longitudinal measures for the *i*^*th*^ subject. We define *t*_1_ < *t*_2_ < *t*_3_ < … < *t*_*D*_ as a set of ordered event times and *Y*_*ij*_(*t*_*i*_) as the time-dependent covariate associated with the individual whose failure time is *t*_*i*_. The risk set *R*(*t*_*i*_) at time *t*_*i*_ is the set of all individuals who are still under study at a time just prior to *t*_*i*_. The partial likelihood based on the hazard function specified (9) can be written as:

9 $$ L\left(\alpha, \beta \right)=\prod \limits_{i=1}^D\left\{\frac{\mathit{\exp}\left(\sum \limits_{k=1}^{m_i}{\beta}_k{Y}_{ik}\left({t}_i\right)+{X_i}^T\alpha \right)}{\sum \limits_{l\epsilon R\left({t}_i\right)}\mathit{\exp}\left[\sum \limits_{k=1}^{m_i}{\beta}_k{Y}_{l k}\left({t}_i\right)+{X_l}^T\alpha \right]}\right\} $$

In most applications, *β*_*k*_ = 0 for intervals which are not under consideration. The estimates can be obtained by maximizing the likelihood specified in (9).

## Simulations

In this section, we carried out a series of simulations to compare the performance of these four methods for modeling longitudinal and survival data described above. The performance of these methods was assessed with Type I error, bias, accuracy, and coverage probabilities for the link parameter. We simulated longitudinal and survival data to resemble data from the Framingham Heart Study. In Table 1, we highlight the simulation model and the required parameters (residual error, random effect means for the longitudinal process, covariance of random effects and coefficients for Age and Sex) for simulating the longitudinal and survival data. The longitudinal trajectories were generated from a linear model adjusting for the age at baseline of the participants. The survival time was generated to depend on the longitudinal measures and a set of covariates (Age at baseline and Sex).

**Table 1:** Simulation Model and Parameters

Exams	(***U***_***i*1**_, ***U***_***i*2**_)	Residual	Age	Sex	Link	Censoring Distribution
6	(4.250, 0.250)	σ^2^ = 0.1161	0.050	−0.500	Varying	Uniform (25, 30)
$$ \mathbf{Random}\ \mathbf{Effects}\ \mathbf{Covariance}\ \mathbf{Matrix}:\kern1em G=\left[\begin{array}{cc}0.29& -0.00465\\ {}-0.00465& 0.000320\end{array}\right] $$						
**Longitudinal Trajectories :** *φ*_*β*_(*t*_*ij*_) *= U*_*i*1_ *+ U*_*i*2_ ∗ t_*ij*_ *+ α ∗ Age*_*i*_						
**Survival Model** **:** *h*(*t*) *= h*_0_(*t*) *exp* {*α*_1_*Age + α*_2_*Sex + γφ*_*β*_(*t*)}						
$$ \mathbf{Survival}\ \mathbf{Time}\ \left(\mathbf{Exponential}\right):\kern1em T=\frac{1}{\left(\ \gamma \ast {U}_{i2}\right)}L\left(\frac{-\gamma \left({U}_{i2}\right)\mathit{\log}(M)}{\lambda exp\left({X}^{\prime}\beta +\gamma \left({U}_{i1}\right)\right)}\right) $$						
$$ \mathbf{Survival}\ \mathbf{Time}\ \left(\mathbf{Weibull}\right):\kern1em T=\frac{1}{\gamma \left({U}_{i2}\ast \frac{1}{\nu}\right)}L\left(\gamma \left({U}_{i2}\ast \frac{1}{\nu}\right)\ast {\left(\frac{-\mathit{\log}(M)}{\lambda exp\left\{{X}^{\prime}\beta +\gamma \left({U}_{i1}\right)\right\}}\right)}^{\frac{1}{\nu }}\right) $$						

In our previous paper on Generating Survival Times with Time-Varying Covariates [29] we provide a detailed algorithm for generating survival times for time-varying Cox Exponential and Weibull models using Lambert’s W function. The simulation requires the specification of the longitudinal measures and the distribution of the survival data. The longitudinal measures can be obtained from a mixed effects model by introducing the random effects as shown in (1) and (2). We focus on a simple linear mixed effect model (LME), which allows individual or subject-specific inference [19] to fit data from longitudinal response process. For the survival data, two independent Weibull distributions were simulated; (i) the survival times that would be observed if the follow-up had been sufficiently long to reach the event and (ii) the censoring mechanism. The survival distribution was generated to depend on the longitudinal measures and a set of covariates in accordance with the model in (5). If the survival time is less than or equal to the censored time, then the event is considered to be observed and the time-to-event equals the survival time; otherwise the event is considered censored and the time-to-event equals the censored time [30]. We assume random non-informative right censoring and employ a uniform distribution for censoring that allows a maximum follow-up time of 30 years. We use the Weibull distribution to generate the survival data. We simulated 1000 independent multivariate datasets consisting of longitudinal measures, time-to-event outcomes, and additional covariates. We present a general formula (see Table 1) which links the survival time of the Cox model and the random effects of the longitudinal model. We use the Weibull distribution to generate survival times from the longitudinal data in the simulation studies by using the Lambert function [29].

We applied the following methods to each of 1000 replicates (10,000 for Type I error): (1) Bayesian semi-parametric joint model (BSJM); (2) Maximum likelihood approach (MLA); (3) Two-step approach (TSA); (4) Time dependent covariate model (TDCM). In the BSJM method several criteria were considered in the MCMC runs including: 1) the number of chains for each run, 2) correlation between successive draws and 3) the length of burn-in time. A total of 101,000 iterations were run with a thinning of 50 and a burn-in of 1000 for 4 chains each, thus providing a sample of 500 iterations per chain. Empirical means and standard deviations for each variable were estimated. The quantiles for each variable are estimated in WinBUGS and used to compute credible intervals. In the Supplemental Material (S5), we present the Bayesian Semi-Parametric Joint Modeling, the initial values as well as the exact prior distributions implemented in the simulations. Two shortcomings of the BSJM are the computing time involved in estimating the parameters and the lack of convergence in some of the MCMC runs.

The variable Sex is considered a fixed covariate at each exam in all the methods. The baseline Age is also included in the model; the data structure is a single row per subject where longitudinal measures, covariates and the overall survival/censoring time are specified for each subject. The statistical analyses were performed using SAS Software (version 9.3; SAS Institute, Cary, NC) and the computing environment R (R Development Core Team, 2012). The Bayesian analysis was conducted in R using WinBUGS version 1.4.3, MRC Biostatistics Unit, Cambridge, UK.

## Results

We compute Type I error for the link parameter to assess all four methods (see Table 2) for a sample size of 100 with 10,000 replicates. The methods appear to provide Type I error rates close to the nominal level (0.050) for both the Exponential and Weibull models with two exceptions. The BSJM shows deflated type I error for both Exponential and Weibull with high censoring rates and elevated Type I error for Weibull with low censoring. The MLA shows elevated Type I errors for Exponential with high censoring (See Supplemental Figure S1).

**Table 2:** Type I Error (*N* = 100, Replicates = 10,000, Link = 0.000)

	**Type I Error (Exponential Distribution)**			
**Censoring**	**TSA**	**MLA**	**BSJM**	**TDCM**
10%	0.057	0.052	0.050	0.056
50%	0.052	0.046	0.040	0.050
90%	0.054	0.078	0.010	0.050
	**Type I Error (Weibull Distribution)**			
**Censoring**	**TSA**	**MLA**	**BSJM**	**TDCM**
10%	0.054	0.054	0.090	0.053
50%	0.057	0.050	0.070	0.059
90%	0.051	0.064	0.030	0.053

*TSA* Two Step Approach, *MLA* Maximum Likelihood Approach, *BSJM* Bayesian Semi-Parametric Joint Modeling, *TDCM* Time Dependent Covariate Modeling

In Table 3 we present the estimates, SEs, coverage probability (CP), bias and mean square error (MSE) for the comparison of the longitudinal effect on survival using the Weibull distribution for *n* = 100. The MLA and BSJM provide lower standard errors and shorter confidence intervals for the link estimate (see Table 3 and Supplemental Figure S2) compared to the other methods. The simulation scenarios with higher censoring rates show higher standard errors and larger confidence intervals. The results suggest the TSA performs best at estimating the link parameter, as it has lower bias and higher coverage probability (CP) compared to the other methods. For example, with 10% censoring and (*γ* = 0.00), the TSA has a bias of 0.000 with a CP value of 95.2%. The TSA provides larger standard errors for the link estimate with larger confidence intervals and CP values close to the nominal level of 95%. The TDCM yields a negative bias with low CP values when there is a strong effect of the longitudinal measure on the outcome.

**Table 3:** Comparison of Longitudinal Effect on Survival (Distribution = Weibull, N = 100, Link = *γ*)

**Scenarios**		**TSA**					**MLA**				
**Censoring**	***γ***	**Estimate**	**SE**	**CP**	**Bias**	**MSE**	**Estimate**	**SE**	**CP**	**Bias**	**MSE**
10%	0.000	0.000	0.247	0.952	0.000	0.125	0.006	0.074	0.955	0.006	0.011
	0.500	0.512	0.251	0.950	0.012	0.128	0.609	0.103	0.848	0.109	0.033
	1.000	1.041	0.266	0.954	0.041	0.142	1.141	0.152	0.904	0.141	0.066
50%	0.000	0.009	0.319	0.950	0.009	0.208	0.006	0.096	0.945	0.006	0.020
	0.500	0.509	0.323	0.954	0.009	0.166	0.572	0.134	0.938	0.072	0.041
	1.000	1.049	0.315	0.945	0.049	0.211	1.190	0.170	0.804	0.190	0.100
90%	0.000	−0.005	0.644	0.948	−0.005	0.868	−0.041	0.188	0.909	−0.041	0.054
	0.500	0.556	0.666	0.947	0.056	0.970	0.588	0.251	0.878	0.088	0.112
	1.000	1.098	0.759	0.940	0.098	1.403	1.243	0.409	0.844	0.276	0.234
		**BSJM**					**TDCM**				
**Censoring**	***γ***	**Estimate**	**SE**	**CP**	**Bias**	**MSE**	**Estimate**	**SE**	**CP**	**Bias**	**MSE**
10%	0.000	0.019	0.073	0.910	0.019	0.014	−0.001	0.188	0.938	−0.001	0.073
	0.500	0.592	0.099	0.920	0.092	0.025	0.336	0.191	0.845	−0.164	0.103
	1.000	1.127	0.145	0.900	0.127	0.053	0.666	0.200	0.595	−0.334	0.191
50%	0.000	−0.004	0.094	0.930	−0.004	0.021	0.013	0.244	0.949	0.013	0.124
	0.500	0.610	0.127	0.930	0.110	0.039	0.354	0.252	0.913	−0.146	0.154
	1.000	1.107	0.156	0.930	0.107	0.059	0.688	0.238	0.712	−0.312	0.214
90%	0.000	0.027	0.191	0.970	0.027	0.076	−0.002	0.494	0.943	−0.002	0.502
	0.500	0.778	0.242	0.933	0.278	0.161	0.373	0.510	0.919	−0.127	0.590
	1.000	1.009	0.269	0.990	0.009	0.116	0.758	0.577	0.907	−0.242	0.838

*TSA* Two Step Approach, *MLA* Maximum Likelihood Approach, *BSJM* Bayesian Semi-Parametric Joint Modeling, *TDCM* Time Dependent Covariate Modeling, *CP* Coverage Probability, *SE* Standard Error, *MSE* Mean Square Error

The performance of the methods in estimating the simulated effects of Age (*α*_1_ = 0.050) and Sex (*α*_2_ =  − 0.500) was also assessed (See Supplemental Figure S3). As the effect of the longitudinal measure on survival becomes stronger, the effect of Age becomes weaker for all the methods except the TSA. The SE’s, CP and confidence intervals tend to be smaller when *n* = 1000, as expected. The results for the sex effect show that all methods provide precise estimates for all the scenarios considered (See Supplemental Figure S4). From the simulation results we see that the standard errors and confidence intervals become larger with higher censoring rates. The sex effect is fairly consistent among the different methods for all simulation scenarios considered.

We also implemented a data generation scheme by Rizopoulos to confirm our results across different settings. We compared the methods using FHS parameters specified in Table 1. The BSJM was not included in this setting due to the computing time involved in estimating the parameters. We used a sample size of 1000 with censoring set at 90% and a link parameter of 0.500. As shown in Table 4, with a residual variance of the longitudinal trajectories (*σ*^2^ = 0.1161), the TSA showed low bias of 0.006 with higher coverage probability 0.952 compared to the other methods. The TSA also provided larger standard errors for the link estimate with larger confidence intervals. The TDCM provided a negative bias of − 0.137 with low CP values of 0.870. The MLA provided the highest estimate in the link parameter with a positive bias of 0.082 and a coverage probability of 0.882. When we applied the above residual variance (*σ*^2^ = 0.1161), the pattern in the results provided similar findings as our data generation scheme. When the residual error was increased (*σ*^2^ = 0.396), to reflect the errors of the joint model by Wulfsohn and Tsiatis [15], we observed a similar trend in the results with the TSA providing the least biased estimate, and nearly 95% coverage, but larger SE’s (see Table 4). The link parameter estimates (0.191) for the TDCM were highly attenuated in this scenario. With a larger residual variance, the TSA showed modest bias (0.452) in the link parameter compared to the scenario with lower residual variance. As seen earlier, the MLA provided larger estimates in the link parameter (0.580) compared to the other methods.

**Table 4:** Rizopoulos Data Generation Scheme (N = 1000, Link = 0.500, Slope = 0.250)

	***σ***^**2**^ ***=*** 0.1161 (FHS), Censoring = 90%			***σ***^**2**^ = 0.396 (Tsiatis), Censoring = 90%		
	TSA	MLA	TDCM	TSA	MLA	TDCM
Estimate	0.506	0.582	0.363	0.452	0.580	0.191
SE	0.214	0.158	0.164	0.241	0.170	0.122
CP	0.952	0.882	0.870	0.945	0.879	0.286
BIAS	0.006	0.082	−0.137	−0.048	0.079	−0.309
MSE	0.092	0.042	0.073	0.119	0.078	0.126

*SE* Standard Error, *CP* Coverage Probability, *MSE* Mean Square Error

## Application to Framingham HEART study

To illustrate the performance of these methods, we examined data from the Framingham Heart Study (FHS) in which lipid measurements and Myocardial Infarction (MI) data were collected over a period of 26 years. The FHS is a widely known longitudinal study that seeks to identify common factors contributing to cardiovascular disease (CVD). Since 1948 three generations of participants have been recruited and followed over the years: Original cohort (recruited in 1948), Offspring (recruited in 1971) and third generation (recruited in 2002). Among the offspring participants, high-density lipoprotein (HDL), low density lipoprotein (LDL) and triglycerides (TG) were measured at fairly similar time intervals over a period of 26 years. The time to myocardial infarction was recorded for each participant, although some subjects were censored by the end of the study period or due to death from other causes. We log transformed the TG measures in our analysis to reduce skewness in TG measures. A total of 2262 subjects with complete data, until event or death, were followed from 1979 to 2005 and data was collected at the start of each exam (see Table 5). The mean age at baseline was 43.3 years. Six exams were considered in the analysis from Exam 2 to Exam 7. The mean triglyceride measures at each exam were calculated with values ranging from 100.49 to 158.70, showing an increased trend from Exam 2 (1979–1983) to Exam 7 (1998–2002). The mean (SD) follow-up time was 22.80 (5.13). The cumulative event rate for the 26-year period was 3.71%. The proportion of female participants was 51%.

**Table 5:** Framingham Heart Study Data (*N* = 2262)

Characteristics	Exam 2	Exam 3	Exam 4	Exam 5	Exam 6	Exam 7
Sample Size – N^a^	2262	2211	2173	2118	2056	1995
Longitudinal Data – Years	1979–1983	1983–1987	1987–1991	1991–1995	1995–1998	1998–2001
Age (Years)	43.32 (9.58)	47.69 (9.60)	51.15 (9.60)	54.80 (9.60)	58.87 (9.54)	61.78 (9.45)
Triglycerides (mg/dL)	100.49 (88.77)	118.80 (123.59)	124.15 (110.18)	154.47 (133.08)	153.08 (114.92)	158.70 (112.49)
Survival Time in Years	4.33 (0.60)	3.43 (0.46)	3.61 (0.46)	4.01 (0.60)	2.87 (0.86)	6.00 (1.62)
Cumulative Event Rate (%)	0.44%	0.88%	1.46%	2.08%	2.39%	3.71%
Sex (% Female)	51.19%					

^a^ Sample sizes reduce at each exam as subjects have events and are censored; Values are Mean (SD) for Continuous Variables

**Fig. 1:**
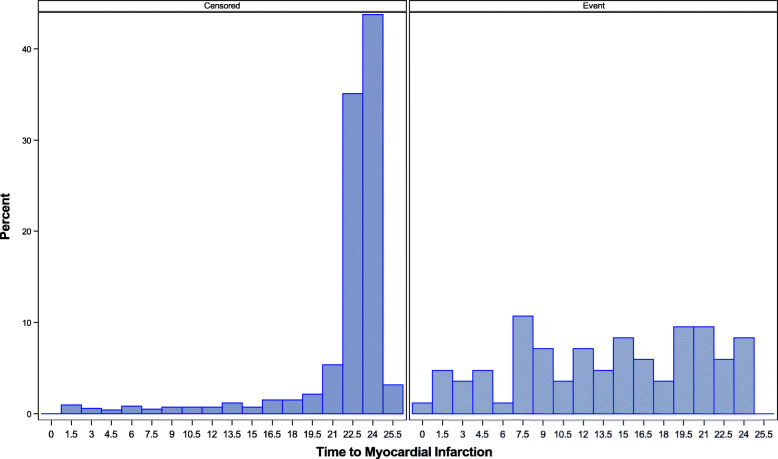
Survival Distribution by Event Occurrence

We fit the FHS data to the models described in Section 2 and characterize the association between the longitudinal measures and time-to-event response. We used log TG at each exam for the longitudinal part of the model assuming a linear trend and survival time measured from exam 2 to MI or loss to follow up (up to 2005). We adjusted for Sex and baseline Age in all the models. In Fig. 1 we show the distribution of time-to-event among the 2262 subjects with complete data. The survival distribution among subjects with events was fairly uniform and the distribution of survival times for censored subjects was skewed to the left with most censoring times occurring at the tail end of the distribution (20–26 years).

In Table 6 we present the estimates for Age, Sex, and the link parameter (*γ*), for each method. The link parameter provides a measure of the association between the longitudinal TG measures and the risk of MI; *γ* is the log hazard ratio for a one unit increase in the log TG at time *t* in the survival model. The link parameter refers to the longitudinal levels at time *t* and does not relate to changes in the longitudinal model over time. The results suggest a higher estimate in the link parameter for the MLA and BSJM (*γ*= 0.9764 and 1.0263). These results are similar to the findings by Wulfsohn and Tsiatis [15] with higher estimates in the joint maximization compared to the two-step approach. The TDCM method shows a lower link estimate compared to the TSA and the joint likelihood methods. The TSA and the MLA provided higher standard errors for the link parameter compared to the other methods. Tsiatis [12] argued that the standard error for the link parameter is greater when using the joint estimation procedure compared to the TSA because the random effects are assumed to be influenced by the uncertainty in the estimated growth curve parameters; thus, more variability is incorporated. We do not see this, however, in these results. The Age effects and standard errors were similar among the methods with estimates ranging from 0.050 to 0.065. The Sex effect was fairly consistent among the different methods ranging from − 1.025 to − 0.999. Using a 0.05 level of significance the Age, Sex and Log of the triglyceride measures were significantly associated with risk of myocardial infarction. The results of all methods suggest that repeated measures of triglyceride levels are significantly associated with the risk of myocardial infarction in the Framingham Heart Study Cohort.

**Table 6:** Jointly Modeling Longitudinal and Survival Data (FHS Data)

	AGE (***α***_**1**_)			SEX (***α***_**2**_)			LogTG (***γ***)		
Methods	Estimate	SE	P	Estimate	SE	P	Estimate	SE	P
TSA	0.0650	0.0120	<.0001	−1.0008	0.2437	<.0001	0.9404	0.2111	< 0.0001
MLA	0.0494	0.0119	<.0001	−0.9992	0.2444	<.0001	0.9764	0.2191	< 0.0001
BSJM	0.0503	0.0121	<.0001	−1.0021	0.2468	<.0001	1.0263	0.1813	< 0.0001
TDCM	0.0560	0.0119	<.0001	−1.0254	0.2435	<.0001	0.6181	0.1741	0.0004

*SE* Standard Error; Parameter Estimates for Age, Sex and Log Triglycerides Specified in Parenthesis

## Discussion

In this paper, we compared longitudinal and survival data methods that link longitudinal trajectories to survival. These methods quantify the link parameter as the association between the current level of a longitudinal process and the survival outcome. We analyzed data from FHS in which triglyceride measurements and Myocardial Infarction (MI) data were collected over a period of 26 years. We used a simulation study to assess the performance of these methods with respect to bias, accuracy, and coverage probabilities. We compared the TSA to the MLA, BSJM and TDCM methods using a derivation of the survival time function for modeling time dependent covariate data.

Based on the simulation studies the TSA, which uses the predicted longitudinal measures, performed best at estimating the link parameter for moderate residual variance. The joint likelihood methods provided upwardly biased estimates in the link parameter, similar to the findings by Wulfsohn and Tsiatis [15]. The TDCM that uses the observed longitudinal measures as time-dependent covariate measures in the survival analysis resulted in underestimation of the true parameters. These results are similar to the findings by Sweeting and Thompson [18]. They recommended the use of a shared random effects model. In most of our models the age effect was attenuated depending on the association of the longitudinal measures on survival. This result was expected as age was associated with the longitudinal measures. The time independent covariates (baseline Age and Sex) were unbiased when there was no association between the longitudinal measures and survival (γ = 0.00). Comparison of the methods in Framingham Heart Study revealed similar patterns.

We implemented a data generation scheme by Rizopoulos in order to confirm our results across the different settings. The results showed similar findings with the two-step approach performing best at estimating the link parameter connecting the longitudinal measures to the event time. For larger residual errors, we found that the TDCM methods had attenuated estimates of the link parameter and the MLA provided upwardly biased estimates. The TSA also yielded biased estimates in the link parameter when there are larger residual errors. These results were similar to the findings by Dafni and Tsiatis [31]. They used different values for the residual error (σ^2^ = 0.32, 0.62, 1.24). In their simulations with larger measurement error, the bias of the estimates based on the observed longitudinal measures increased dramatically in the positive direction. They indicated that the two-step model yielded parameter estimates that were somewhat biased towards the null (for larger residual errors). We explored several other scenarios not presented in this paper where we varied the residual error in the longitudinal measures. Our results show that with low residual errors in the longitudinal measures, the TSA provides results similar to time dependent covariate methods that use the observed longitudinal measures. We interpreted this to mean that a small residual error results in low measurement error and the observed values are comparable to the predicted values.

One limitation of our study is the linearity assumption in the longitudinal measures. The trajectory can be modeled in a linear form [4] or quadratic form [12]. Splines and other time series forms can also be implemented to better capture the trajectory of the longitudinal measures, but the trade-off is the complexity of the model and interpretability. Further, the creation of the simulated data depends on the linear model for the longitudinal data. Exploration of non-linear models for the longitudinal data is a topic of future research. The simulation data generation scheme was also based on the two-step approach; this may provide more precise estimates when analyzing the simulated data using the two-step model. Another limitation is the number of time points used for the longitudinal measures. With more exam visit time-points the LME model becomes computationally intensive. In addition, the use of distributions other than the Exponential and Weibull is indispensable in investigating the characteristics of the Cox proportional hazard model. There is the need for the use of empirical distributions to handle flexibly parameterized proportional hazard models [32]. Despite these limitations this paper strengthens the current knowledge on methods for jointly modeling longitudinal and survival data.

## Conclusion

Traditional methods, such as TDCM that use observed data, tend to provide downwardly biased estimates towards the null. The TSA and joint models provide better estimates, although a comparison of these methods may depend on the underlying residual variance. Hence, an avenue for future exploration is to evaluate the degree of attenuation in the link parameter by the magnitude of the residual variance. Joint modeling for longitudinal and survival data has also recently received attention in statistical genetics research. In genome wide association studies and gene expression studies, longitudinal and survival measures are often collected over time. The development of new methods to handle these high dimensional data is essential. Binary longitudinal measures, multiple survival endpoints and missing data analyses are also important areas for further investigation using the methods for jointly modeling longitudinal and survival data.

## Supplementary Information

**Additional file 1: S1:** Type I Errors for Link (Exponential and Weibull Distribution, *N* = 100). **S2:** Estimates and Confidence Intervals for Link (Weibull Distribution, N = 100). **S3:** Estimates and Confidence Intervals for Age (Weibull Distribution, N = 100). **S4:** Estimates and Confidence Intervals for Sex (Weibull Distribution, N = 100). **S5:** Bayesian Semi-Parametric Joint Modeling Exact Prior Distributions

## Data Availability

The data used in this study are available at NIH BioLINCC (https://biolincc.nhlbi.nih.gov/home/). They can also be provided to interested researchers on written request to FHS. Request for FHS data may be done by submitting a proposal through the FHS web-based research application. A catalogue of the FHS data repository may be accessed through the FHS website: www.framinghamheartstudy.org/researchers/description-data/

## Abbreviations

BSJM: Bayesian Semi-Parametric Joint Modeling
CP: Coverage Probability
LME: Linear Mixed Effects
MLA: Maximum Likelihood Approach
TDCM: Time Dependent Covariate Modeling
TSA: Two Step Approach
MSE: Mean Square Error
