# Supplementary figures and images for: Revisiting methods for modeling longitudinal and survival data: Framingham Heart Study
Source: BMC Med Res Methodol. 2021 Feb 10;21:29. doi: 10.1186/s12874-021-01207-y (PMC7876802; doi:10.1186/s12874-021-01207-y)

**S1**


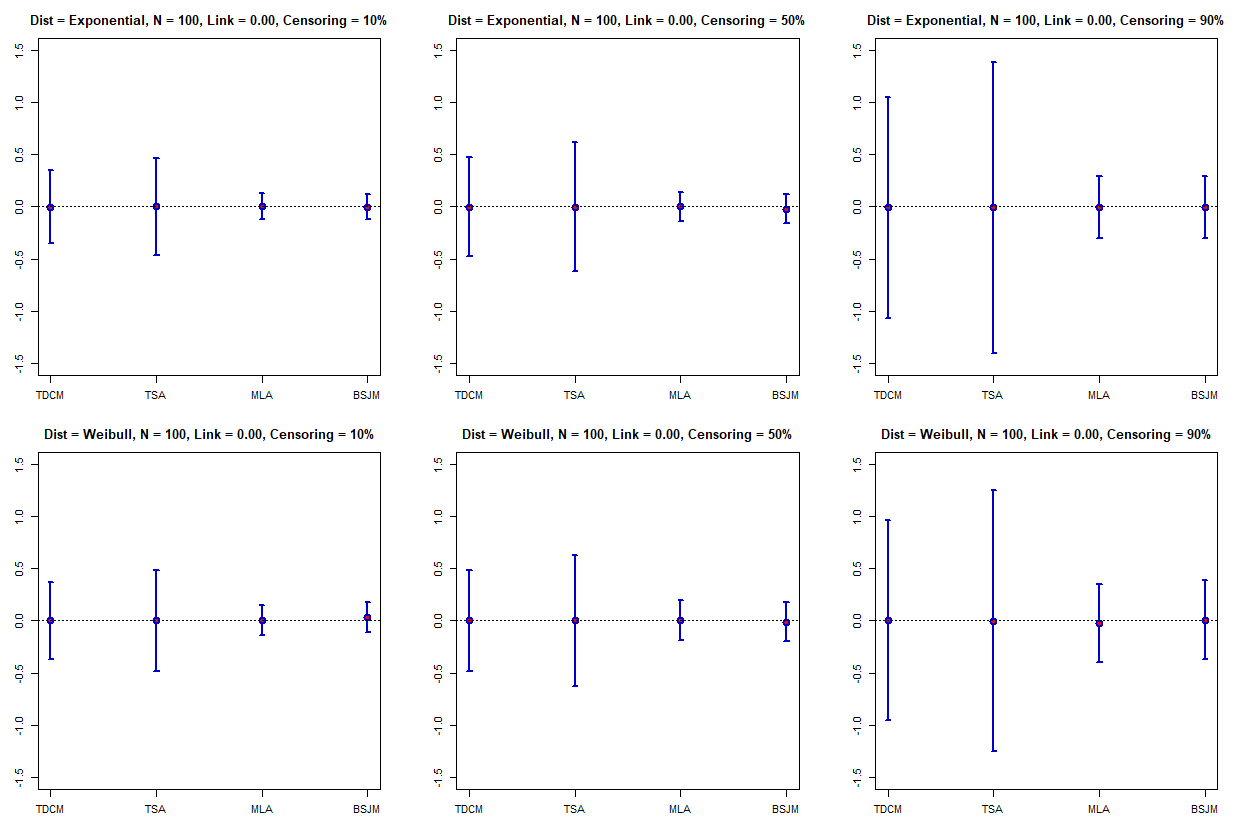


**S2**


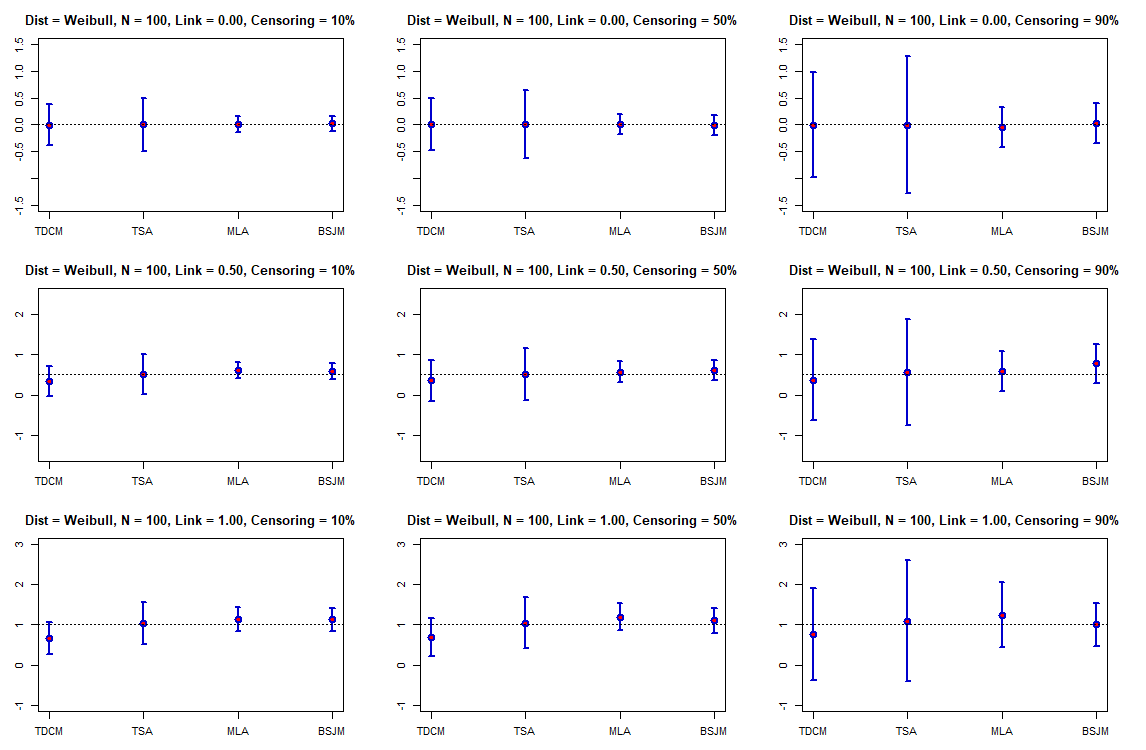


S3


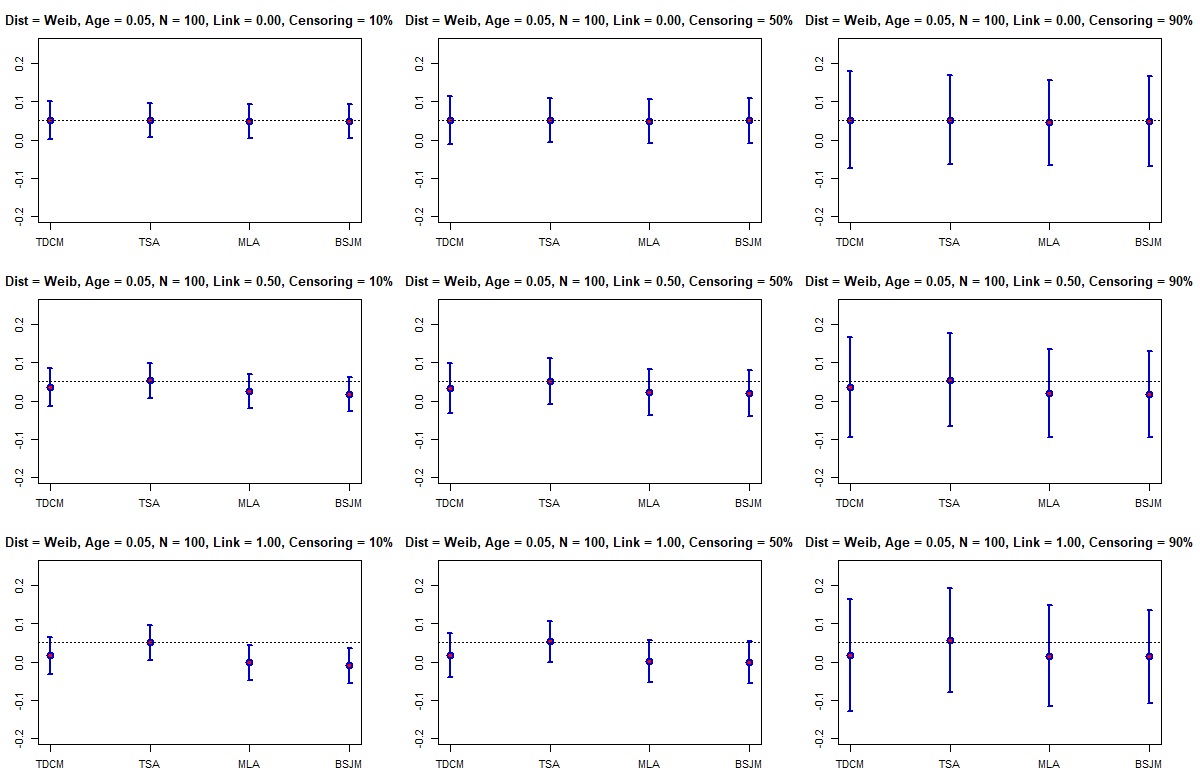


S4


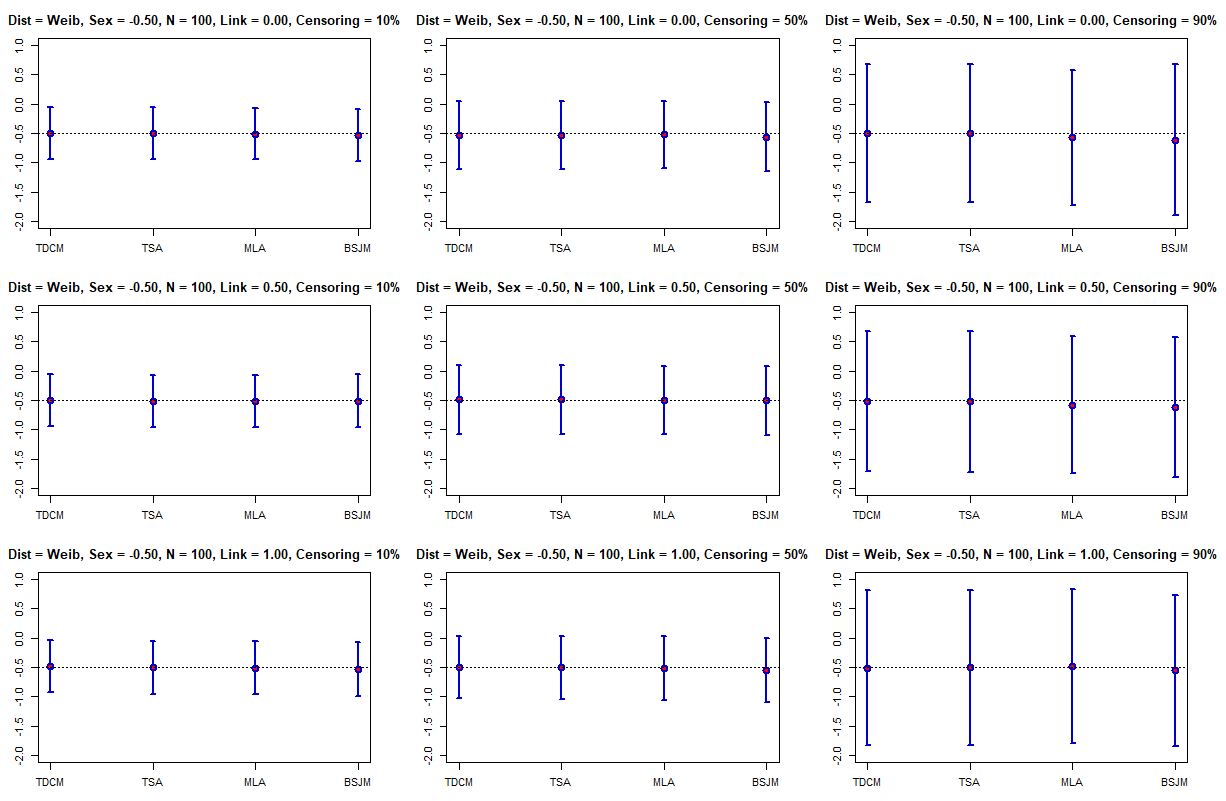


**
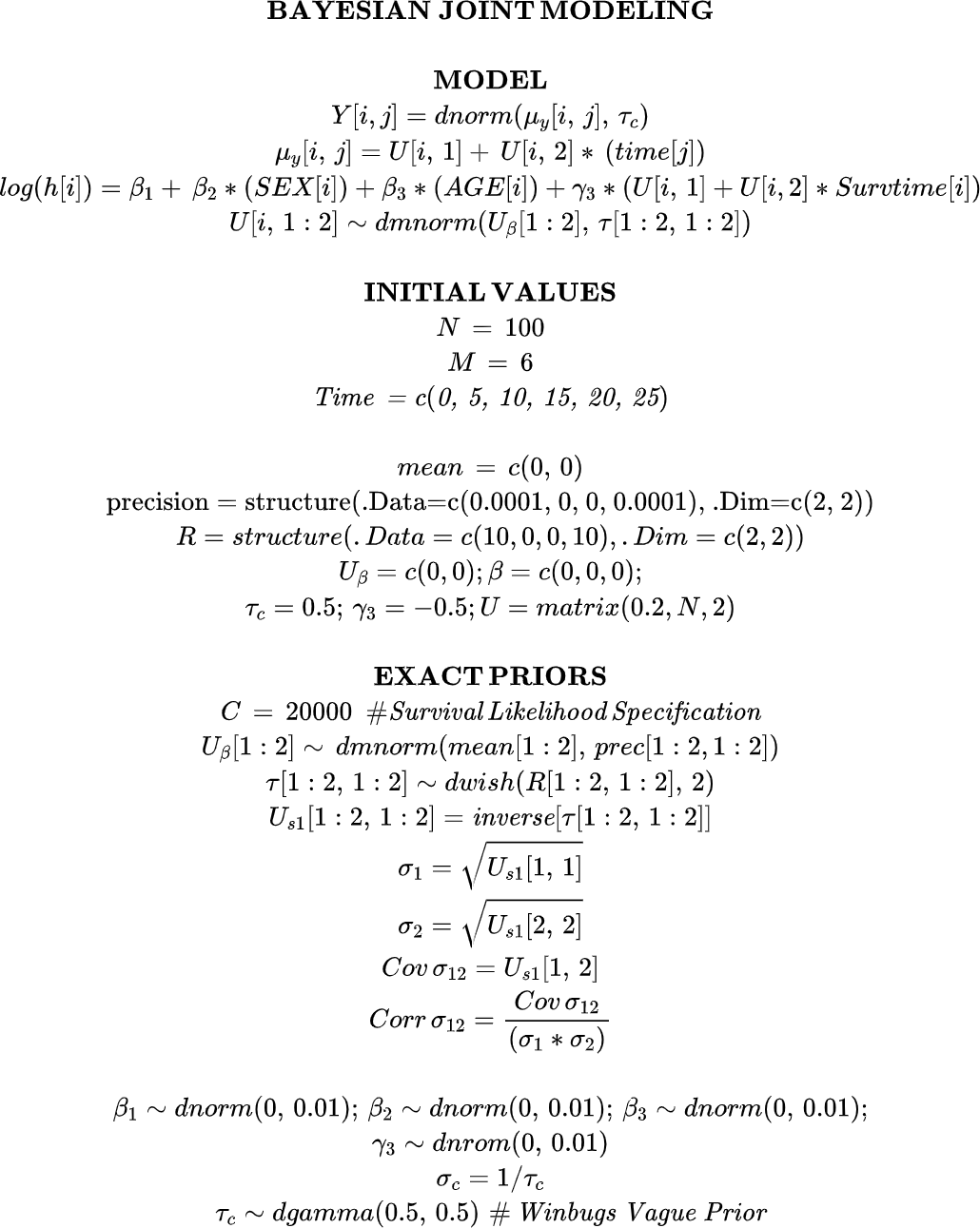
**

Supplement: Supplementary file 1 — Additional file 1: S1: Type I Errors for Link (Exponential and Weibull Distribution, N = 100). S2: Estimates and Confidence Intervals for Link (Weibull Distribution, N = 100). S3: Estimates and Confidence Intervals for Age (Weibull Distribution, N = 100). S4: Estimates and Confidence Intervals for Sex (Weibull Distribution, N = 100). S5: Bayesian Semi-Parametric Joint Modeling Exact Prior Distributions [file 12874_2021_1207_MOESM1_ESM.docx]
